# Auxin Involvement in Ceratopteris Gametophyte Meristem Regeneration

**DOI:** 10.3390/ijms242115832

**Published:** 2023-10-31

**Authors:** Kelley A. Withers, Athena Kvamme, Christopher E. Youngstrom, Rebekah M. Yarvis, Rachel Orpano, Gabriel P. Simons, Erin E. Irish, Chi-Lien Cheng

**Affiliations:** Department of Biology, University of Iowa, Iowa City, IA 52242, USA; kelley-withers@uiowa.edu (K.A.W.); athena-kvamme@uiowa.edu (A.K.); christopher-youngstrom@uiowa.edu (C.E.Y.); rebekah-yarvis@uiowa.edu (R.M.Y.); rachel.orpano27@gmail.com (R.O.); gabriel-simons@uiowa.edu (G.P.S.); erin-irish@uiowa.edu (E.E.I.)

**Keywords:** fern apical cells, auxin gradient, laser ablation, WOXB, TAA2, lateral meristem

## Abstract

Growth and development of the Ceratopteris hermaphroditic gametophytes are dependent on cell proliferation in the marginal meristem, which when destroyed will regenerate at a new location on the body margin. We established a laser ablation method to destroy a single initial cell in the meristem. Ablation caused the cessation of cell proliferation accompanied by the disappearance of the expression of an auxin synthesis gene (*CrTAA2*) and a cell proliferation marker gene (*CrWOXB*). New meristem regeneration occurred within a predictable distance from the original two days post-ablation, signified by cell proliferation and the expression of *CrTAA2*. Treatment with the naturally occurring auxin indole-3-acetic acid (IAA), synthetic auxin 2,4-dichlorophenoxyacetic acid (2,4-D), or the transport inhibitor naphthylphthalamic acid (NPA) altered positioning of the original marginal meristem toward the apex of the gametophyte. IAA altered positioning of the regenerated meristem after damaging the original meristem. A model of auxin involvement in the positioning of the marginal meristem in Ceratopteris is presented to encompass these results.

## 1. Introduction

The life cycle of a land plant alternates between two multicellular stages, the diploid sporophyte and the haploid gametophyte. In vascular plants, the dominant sporophyte maintains growth and development throughout its lifespan by maintaining cell divisions in meristems. These meristems are mainly located at the shoot tip, root tip, and vasculature. The gametophytes’ ability to grow and develop differs greatly among vascular plants. On one extreme, gametophytes of flowering plants do not have meristems, are extremely reduced, and are sporophyte dependent. On the other extreme, gametophytes of seed-free vascular plants (lycophytes and ferns) contain meristems, are free-living, and are capable of photosynthesis during growth and development (see a recent review by Fouracre and Harrison [1]).

Meristems of small and single-cell-layered fern gametophytes offer an attractive system for investigating the behavior of meristems during development [2,3,4,5]. Fern gametophyte meristems can be classified into two types, the apical cell (AC)-based meristem and the AC-independent multicellular meristem [6]. In the model homosporous fern Ceratopteris (*Ceratopteris richardii*), the “apical meristem” (defined below) supports the early development of gametophytes. A second meristem, the marginal (also called lateral or notch) meristem, appears later in development of the hermaphrodite and replaces the apical meristem as the source of cell proliferation in the hermaphrodite (reviewed by Banks [7]). Male gametophytes do not form a marginal meristem and exhibit determinant growth. More recently, this developmental pattern has been examined in more detailed analyses [8,9,10]. During germination, a spore divides asymmetrically to give rise to the small AC and the rhizoid-producing basal cell; the AC divides again to form a three-celled, one-dimensional protonema. Next, the AC quickly loses its morphological identity, and all cells have a similar division rate during the formation of a two-dimensional, tongue-shaped prothallus [8]. The apical domain of the prothallus described by Bartz and Gola [8] is also where the transcription factor *CrLEAFY* is expressed and has been called the “apical meristem” [11], which we have adopted here.

Immediately following the tongue-shaped prothallus stage (stage three by Bartz and Gola [8]), the hermaphrodite develops a marginal meristem on one side of the prothallus that replaces the apical meristem. Different methods have been used to examine the initiation and development pattern of the marginal meristem, yielding different results. Using fixed samples for lineage analysis in combination with observing cell division activity, Bartz and Gola [8] detected four distinctive cells (resulting from sequential anticlinal divisions) at the margin of the nascent marginal meristem while other cells of the tongue-shaped prothallus still underwent both anti- and peri-clinal divisions; eventually, a single “initial cell” was identified at the center of the marginal meristem. The authors further grouped cells in a mature hermaphrodite into three lineages that were defined in stage three. In another study, time-course confocal imaging of live gametophytes and computational quantification to track cell lineages of the hermaphrodite determined approximately three progenitor cells (hereafter, marginal initials) which contributed to meristem initiation and proliferation, while a singular initial cell could not be identified (Geng et al. [5]). Cell division occurred more frequently in these marginal cells than inner cells of the meristem, and the occurrence was independent of cell lineages.

A logical approach to studying meristem behavior is to destroy the existing one and observe the effects at this site and any new meristematic activity elsewhere on the gametophyte. The marsh fern *Thelypteris palustrus* has an AC-based meristem which, upon mechanical destruction of the AC, is able to generate a new AC and meristem in a new region within five days [12]. Thus, the author hypothesized that the single *T. palustrus* AC inhibits all other cells of the thallus from becoming apical cells. Support for the naturally occurring auxin indole-3-acetic acid (IAA) as the inhibitor of fern AC identity comes from studies by Albaum [13] in *Pteris longifolia*, which has a multicellular meristem. When a *P. longifolia* gametophyte is cut in half, the apical part containing the meristem continues the development path of a whole gametophyte whereas the basal part regenerates adventitious prothalli from the cut side. The application of IAA to the cut side of the basal part inhibits prothallus regeneration [13]. Recently, Geng et al. [5] demonstrated that microneedle ablation of a few cells of the Ceratopteris marginal meristem is sufficient to cause new meristem regeneration. After ablation, cell divisions surrounding the destroyed area decrease. In this report, we established a laser ablation method that generated sufficient material to allow for a more detailed analysis of meristem regeneration in the fern gametophytes. We used laser ablation to target single marginal initials at the Ceratopteris marginal meristem and tested whether auxin affects the regeneration of new meristems. We also described gene expression differences between the original and regenerated meristems of the gametophyte by utilizing the cell proliferation marker *WUSCHEL-RELATED HOMEOBOX B (CrWOXB)* [14] and *CrTAA2,* a homolog of auxin biosynthetic gene *TRYPTOPHAN AMINOTRANSFERASE OF ARABIDOPSIS 1* (AtTAA1) [15,16].

## 2. Results

### 2.1. Laser Ablation of a Single Marginal Meristem Initial Cell Triggers Regeneration

**Figure 1:**
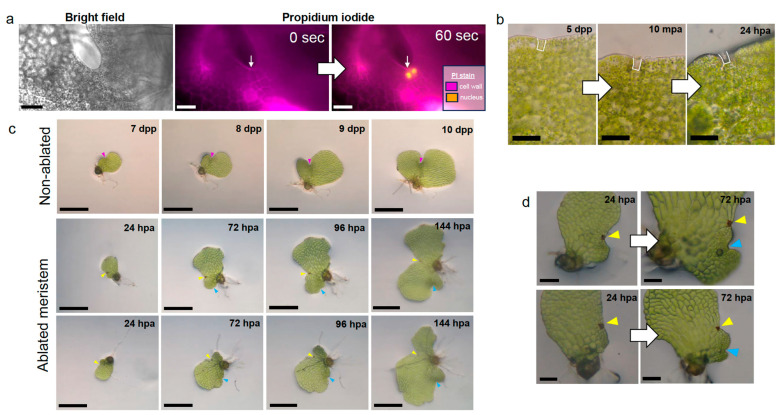
Laser ablation of a marginal meristem initial triggers formation of a new meristem. (**a**) Bright field greyscale image of a 10-dpp hermaphrodite marginal meristem with corresponding fluorescence micrographs at 0 s (before ablation, cell wall emission at 561 nm, magenta hue) and 60 s post-ablation (cell wall emission at 561 nm, magenta hue; nucleus-bound 615 nm, orange hue) of a marginal meristem initial by exposure to high-intensity laser. White arrows indicate the targeted marginal initial cell. (**b**) Bright field images of a 5-dpp hermaphrodite before (**left**), ten minutes after (**middle**) laser ablation, and 24 h post-ablation (**right**). White outline marks targeted marginal initial cell without (**left**) or with (**middle**,**right**) lysed cell contents. (**c**) Representative time-course images of non-ablated (7- to 10-days post-plating (dpp)) and ablated (24- to 144-h post-ablation (hpa)) hermaphrodite gametophytes. Magenta arrowheads denote the original marginal meristem position, yellow arrowheads mark the site of laser ablation damage, and cyan arrowheads mark the position of the regenerated meristem. (**d**) Representative bright field microscope images of hermaphrodite prothalli before regeneration is visible, 24-hpa, and after the regenerated meristem develops the first archegonium, 72-hpa. (**a**,**b**) Scale bar = 50 μm. (**c**) Scale bar = 0.5 mm. (**d**) Scale bar = 100 μm.

Initiation of the marginal meristem in hermaphroditic gametophytes is detected by the presence of marginal cells on one side of the prothallus which undergo anticlinal divisions to form thin rectangular cells [8] and have higher proliferative activity than inner cells [5]. We first tested our ability to destroy individual marginal cells by ablating targeted cells of propidium iodide-stained gametophytes with a high intensity laser. A healthy unablated gametophyte restricted propidium iodide to the cell walls, but after ablation, the targeted cell had a high intensity propidium iodide signal localized to the nucleus (Figure 1a). This indicates that laser ablation compromises the integrity of the targeted cell. We observed that the unablated neighboring cell was also capable of taking up propidium iodide into the nucleus within one minute. To confirm that the ablation of an individual cell did not also disrupt the integrity of adjacent marginal cells, we used bright field microscopy to visualize the meristem area of gametophytes prior to and ten minutes after laser ablation, as well as 24 h later. Visible cell lysate from the ablated cell was indicative of successful targeting and occurred without mechanical damage to the adjacent cells, which retained their cell contents and remained green (Figure 1b), therefore illustrating our capability to disrupt individual marginal cells.

Using our precise ablation technique, we ablated the marginal meristem initial cell, as defined by Bartz and Gola [8], of 5-days post-spore plating (dpp) gametophytes to assess their development after targeted cell loss. Unablated individuals were grown as controls for gametophyte development: marginal meristems exhibit indentation between the two lobes at 7-dpp, archegonium development at the meristem begins at 8-dpp, and the second lobe continues to expand from 9- to 10-dpp to form the typical chordate shape. (Figure 1c, top row). Ablated gametophytes were imaged every 24 h post-ablation (hpa), with a new site of proliferative activity becoming apparent between 24- and 72-hpa (Figure 1c, second and third rows). The new region of dense cells became a regenerated meristem complete with two lobes, and the production of archegonia normally seen close to the marginal meristem of non-ablated gametophytes (Figure 1c,d). The regeneration observed here is similar to that described by Geng et al. [5] after microneedle ablation of multiple meristem cells but was initiated after ablation of a single meristem cell. The regeneration phenotype was reproducible across batches of gametophytes and was specific to marginal initial cell ablation, as gametophytes with ablation behind the meristem or at the margin away from the meristem continued to proliferate at the original meristem location comparably to the non-ablated controls (Figure S1).

### 2.2. Meristem Regeneration Occurs within a Predictable Range from Site of Damage

**Figure 2:**
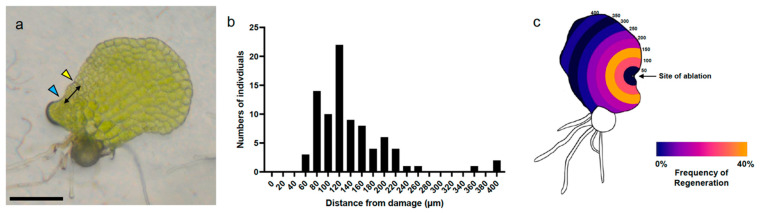
Meristem regeneration occurs at a predictable distance from the site of laser ablation. (**a**) Schematic of RMD measurements from the site of laser ablation to the origin of the regenerated meristem. Yellow arrowhead marks the laser ablation site and cyan arrowhead marks the position of the regenerated meristem. Black arrows span between the two locations, denoting the distance from damage. (**b**) Frequency histogram of RMD measurements collected from ablated gametophytes grown on basal media (*n* = 85). (**c**) Heatmap of regenerated meristem frequency in 50 μm intervals, illustrated on a representative gametophyte with concentric rings from 0–400 μm, centered on the site of ablation (marked with a black arrow). Scale bar = 0.25 mm.

Laser ablation of individual meristem initial cells triggered meristem regeneration at a new location on the Ceratopteris gametophyte body. Therefore, we sought to understand how the new meristem location is designated. Using images of 48-hpa gametophytes (*n* = 85), we measured from the site of ablation to the center of the regenerated meristem and defined this distance as the regenerated meristem distance (RMD; see Methods for details) (Figure 2a). The distribution of the RMD values (Figure 2b) spanned from 64 to 399 μm and was skewed positively in shape, with a median distance of 122 μm. Infrequently, the new meristem regenerated over 200 μm away from the ablation site in cells at the opposite margin. Meristem regeneration was never observed at a distance less than 64 μm, suggesting the existence of a positional signal responsible for initiation of a new meristem at a preferable distance away from the original damaged meristem.

### 2.3. Ceratopteris Auxin Biosynthesis Gene Expression Localizes to Marginal or Regenerated Meristems

Previous work by Gregorich and Fisher [2] showed that Ceratopteris gametophytes grown on synthetic auxin compounds (NAA and 2,4,5-T) exhibit delayed lateral meristem formation. Auxin has also been implicated in the response of *Marchantia polymorpha* gametophytes to removal of the apical meristem region [17], making it a suitable hormone to investigate with our regeneration system. Canonical biosynthesis of auxin proceeds through two steps: the conversion of L-tryptophan to indole-3-pyruvate (IPA) by TRYPTOPHAN AMINOTRANSFERASE OF ARABIDOPSIS (TAA) transaminases, followed by the conversion of IPA to indole-3-acetic acid (IAA) by YUCCA (YUC) flavin monooxygenases [18].

To detect regions of the gametophyte with higher transcript levels of auxin biosynthesis genes, we first identified seven homologs of *TAA* in the Ceratopteris genome. The *CrTAA* homologs with the most similarity to Arabidopsis *TAA1* were chosen based on a sequence alignment of conserved tryptophan aminotransferase and transmembrane domains between the known Arabidopsis amino acid sequence and Ceratopteris predicted protein sequences, as well as our constructed phylogeny with 61 whole protein sequences from 17 land plant representatives (Figure S2a, Table S1). CrTAA1 (Ceric.10G039800), grouped in the top clade along with AtTAA1 and AtTAR1, was the only Ceratopteris gene in this clade. In the bottom clade, all other identified fern TAA/TAR sequences formed a sister clade with angiosperm proteins such as AtTAR3 and AtTAR4. CrTAA2 (Ceric.25G013500) and CrTAA3 (Ceric.29G002600) were chosen as representatives from this fern-specific clade.

Next, we quantified the expression changes of our chosen Ceratopteris TAA genes, *CrTAA1*, *CrTAA2*, and *CrTAA3*, along with a single Ceratopteris *YUCCA* homolog, *CrYUC2-3* (Ceric.05G083300), using RT-qPCR in 7-, 10-, and 13-dpp gametophyte tissues (see Table S2 for the primer sequences). We reasoned that a gene expressed higher during marginal meristem establishment between 5 and 7-dpp would be the best candidate gene for determining regions of auxin biosynthesis activity. *CrTAA2* showed the most dynamic expression of the auxin biosynthesis genes examined, with significantly higher expression at 7-dpp in comparison to both 10-dpp and 13-dpp (Figure S3a). *CrTAA1* maintained expression between 7-dpp and 10-dpp, with a significant decrease at 13-dpp (Figure S3a). Expression of *CrTAA3* and *CrYUC2-3* did not significantly change between the timepoints examined (Figure S3a). Due to the dynamic expression of *CrTAA2* during gametophyte growth, we chose to proceed with expression localization for this gene.

**Figure 3:**
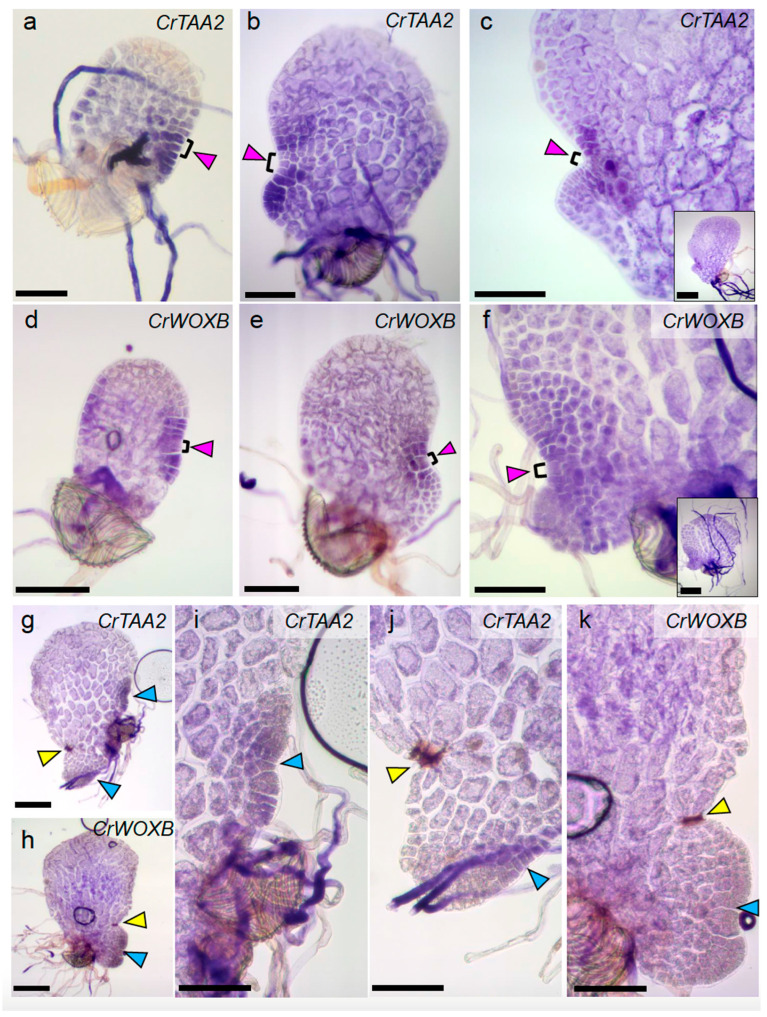
Localization of *CrTAA2* and *CrWOXB* expression in non-ablated and ablated gametophytes. (**a**–**c**,**g**,**i**,**j**) Whole mount in situ hybridization of the *CrTAA2* and (**d**–**f**,**h**,**k)** *CrWOXB* antisense probes in gametophyte tissue (black brackets indicate marginal initial cells): (**a**,**d**) 5-dpp gametophyte with newly formed marginal meristem, (**b**,**e**) 7-dpp hermaphrodite with marginal meristem and proliferating second lobe, (**c**,**f**) 9-dpp hermaphrodite with continued cell proliferation at the marginal meristem and archegonium development (inset shows whole gametophyte), (**g**–**k**) Gametophytes 72-h post-ablation. (**g**,**i**,**j**) Presence of *CrTAA2* expression at the regenerated meristem and absence at the site of ablation. (**i**–**k**) Magnified from same individuals shown in (**g**,**h**), respectively. (**h**,**k**) *CrWOXB* expression is absent at both the regenerated meristem and the site of ablation. (**a**–**k**) Scale bars = 0.1 mm. (**c**,**f**) Inset scale bars = 0.2 mm. Magenta, yellow, and blue arrowheads point at the original, abated, and regenerated meristems, respectively.

The full-length coding sequence for *CrTAA2* was cloned and used to generate labeled RNA probes for whole mount in situ hybridization in gametophyte tissues collected 5-, 7- and 9-dpp (see Table S2 for primer sequences). The gene *WUSCHEL-RELATED HOMEOBOX B* (*CrWOXB*), a transcription factor which promotes cell proliferation in the gametophyte [14], was used as a marker for the marginal meristem as it expresses in this region at all timepoints examined. In 5-dpp gametophytes, expression of *CrTAA2* and *CrWOXB* are detected in a subset of the marginal cells that will generate the marginal meristem (Figure 3a,d; see sense images in Figure S4a–j). Localization of *CrTAA2* persisted at the marginal meristem at 7-dpp, with one surprising change: the marginal initial cells with the highest proliferative activity, as described by Geng et al. [5], appeared to have less expression of *CrTAA2* than the surrounding marginal cells (Figure 3b), a pattern we consistently observed in gametophytes that had developed to the point of secondary lobe formation. This pattern of expression continued in the 9-dpp samples, with additional expression of *CrTAA2* in newly produced archegonia (Figure 3c). The gene–expression distinction between marginal initials and other marginal cells of the meristem has been observed previously with *CrWOXB* at 10-dpp [14], which we were also able to observe in the current experiments with CrWOXB at 7-dpp and 9-dpp (Figure 3e,f). In situ hybridization of *CrYUC2-3* in 7-dpp gametophytes revealed the localization of *CrYUC2-3* transcripts to the marginal meristem (Figure S3b,c), confirming that the pattern of *CrTAA2* expression is likely to mark areas with auxin biosynthesis activity. Taken together, our expression data showed that *CrTAA2* and *CrWOXB* share similar expression patterns at the marginal meristem at 5-, 7-, and 9-dpp. Less signal of *CrTAA2* hybridization was observed in the marginal initials in comparison to adjacent marginal cells of the meristem in gametophytes older than 5-dpp, which may indicate differences in auxin biosynthesis gene expression between the marginal initial cells and neighboring cells within the marginal meristem.

We were curious to see if a shift in the expression domain of *CrTAA2* from the original meristem to the site of regeneration occurred in gametophytes post-ablation during the establishment of a new proliferative region. To examine this, we harvested gametophytes ablated at 5-dpp after 72-hpa of recovery on agar media and performed whole mount in situ hybridization. Regenerated meristems appeared morphologically similar to non-ablated 7-dpp samples and showed the same, albeit weaker overall, expression of *CrTAA2* in the marginal cells of the new meristem (Figure 3g,i). Meanwhile, *CrTAA2* expression disappeared at the ablation site (Figure 3j). We were also able to eliminate *CrWOXB* expression at the original meristem by ablation (Figure 3h,k). Therefore, single cell ablation was able to change the expression of the marginal meristem markers at the original meristem exemplified by *CrTAA2* and *CrWOXB*. Interestingly, the strong expression of *CrWOXB* observed at the meristem of non-ablated gametophytes did not appear at the regenerated meristem 72-hpa of ablated gametophytes (Figure 3k). The relocation of *CrTAA2* expression after ablation suggests an important role for auxin biosynthesis in maintaining proliferative capacity in the gametophyte meristem.

### 2.4. Exogenous Auxin and Auxin Inhibitors Modulate Gametophyte Morphology

**Figure 4:**
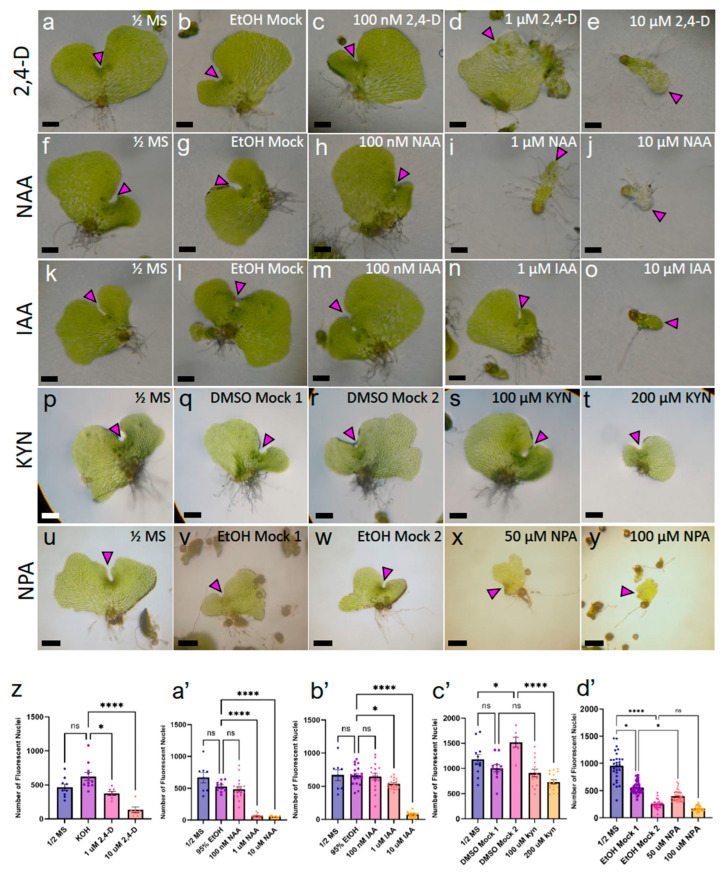
Hermaphrodites treated with auxin chemicals exhibit decreases in cell proliferation and morphological defects. (**a**–**e**,**z**) An 11-day dosage with 2,4-dichlorophenoxyacetic acid (2,4-D) on gametophyte growth. (**f**–**j**,**a’**) An 11-day dosage with 1-napthaleneacetic acid (NAA). (**k**–**o**,**b’**) An 11-day dosage with indole-3-acetic acid (IAA). (**p**–**t**,**c’**) A 12-day dosage with L-kynurenine (KYN). (**u**–**y**,**d’**) A 14-day dosage with naphthylphthalamic acid (NPA). (**a**,**f**,**k**,**p**,**u**) Basal media controls. Solvent mock representatives: (**b**) 95% EtOH mock for 2,4-D, (**g**) 95% EtOH mock for NAA, (**l**) 95% EtOH mock for IAA, (**q**,**r**) DMSO mocks for 100 μM and 200 μM KYN, respectively, (**v**,**w**) 95% EtOH mocks for 50 μM and 100 μM NPA, respectively. 2,4-D dosage of (**c**) 100 nM, (**d**) 1 μM, and (**e**) 10 μM. NAA dosage of (**h**) 100 nM, (**i**) 1 μM, and (**j**) 10 μM. IAA dosage of (**m**) 100 nM, (**n**) 1 μM, and (**o**) 10 μM. KYN dosage of (**s**) 100 μM and (**t**) 200 μM. NPA dosage of (**x**) 50 μM and (**y**) 100 μM. (**z**–**d’**). Quantification of cell number from individuals grown on auxin chemicals, via DNA staining with Hoechst 33,342 (*n* ≥ 6, mean ± SEM, (**z**,**d’**) Kruskal–Wallis with Dunn’s post-hoc test, (**a’**,**c’**) one-way ANOVA with Sidak’s post-hoc test, or (**b’**) Dunnett’s post-hoc test; ns, not significant; *, *p* ≤ 0.05; ****, *p* ≤ 0.0001). Arrowhead pointing at marginal meristem, scale bars = 0.25 mm.

Areas of local auxin biosynthesis have been implicated in major development processes of angiosperms and play a role in the environment-triggered modulation of growth (recently reviewed by Zhao [19]). Our discovery of broad *CrTAA2* expression in 5-dpp gametophytes and specific localization to the marginal meristem of 7-dpp hermaphrodites (Figure 3) in combination with its expression pattern during meristem ablation and regeneration led us to hypothesize that auxin plays a role in marginal meristem development. To test this, we examined the effect of the exogenous synthetic auxins 2,4-dichlorophenoxyacetic acid (2,4-D) and 1-Napthaleneacetic acid (NAA), the naturally occurring auxin IAA, an auxin biosynthesis inhibitor L-kynurenine (KYN), and an auxin transport inhibitor N-1-naphthylphthalamic acid (NPA) on gametophyte morphology. Gametophytes were grown on a concentration series of each chemical incorporated into agar media (Figure 4a–y), with tissue samples collected after the basal media-grown hermaphrodites had reached sexual maturity. Gametophyte cell number was measured for each treatment to determine the effect of the auxin chemicals on cell proliferation (Figure 4z–d’).

A dosage of 10 μM 2,4-D was able to significantly reduce the number of cells per gametophyte (133 cells ± 105) compared to the basal media control (464 cells ± 146) and solvent mock (623 cells ± 192) (Figure 4a,b,e,z). 10 μM 2,4-D also severely altered the morphology of the gametophytes, resulting in over-proliferation of rhizoids and marginal meristem formation on the apical end instead of laterally, and no antheridia or archegonia (Figure 4e). 1 μM 2,4-D had a weaker effect on the number of cells; gametophytes produced fewer cells (375 cells ± 80) compared to the solvent mock (623 cells ± 192) (Figure 4b,d,z), although the chordate shape was distorted by elongation of the oldest cells and a lack of extension in the new lobe. Like the 10 μM 2,4-D dosage, 1 μM 2,4-D increased rhizoid production, but it was limited to the basal side. In contrast, 1 μM 2,4-D did not inhibit gametangium formation (Figure 4d). The effect of 100 nM 2,4-D was negligible (Figure 4c).

NAA had a more pronounced effect on gametophyte development than 2,4-D. 100 nM NAA caused the older cells of the gametophyte body to enlarge and promoted rhizoid production, without altering the number of cells produced (486 ± 166) in comparison to the solvent mock (527 cells ± 83) (Figure 4g–h,a’). Growth on 1 μM NAA stunted gametophyte development; the prothalli were small and elongated in shape, less than 10 cells in width, with abnormal rhizoid production along the entire margin (Figure 4i). 1 μM NAA promoted apical-side marginal meristem development, a phenotype previously reported [2] and similar to what we observed with 10 μM 2,4-D (Figure 4e,i). 1 μM NAA resulted in a drastic decrease in cell number (54 ± 13) in comparison to the solvent mock (527 cells ± 83) (Figure 4a’). 10 μM NAA caused the gametophytes to arrest at a stage similar to basal media 3-dpp, with cells that were enlarged and looked pale, with rhizoid-like protrusions (Figure 4j). No marginal meristems were formed, and the number of cells produced was much lower (65 ± 35) than the solvent mock (527 cells ± 83) (Figure 4a’).

IAA, a naturally occurring auxin, required a higher concentration to induce the same morphological changes as 2,4-D and NAA. Gametophytes in 100 nM IAA had a comparable number of cells (645 ± 199) to the solvent mock (662 ± 142) and no noticeable changes in shape or meristem placement (Figure 4l–m,b’). 1 μM IAA significantly decreased the number of cells (538 ± 72) compared with the solvent mock (662 ± 142) but still retained a proper chordate shape and gametangium development (Figure 4n,b’). 10 μM IAA was the only treatment to produce a noticeable effect on gametophyte development, with smaller and more elongated prothalli (Figure 4o). 10 μM IAA drastically decreased the number of cells produced (76 ± 37) (Figure 4b’). Apical-side positioning of the marginal meristem was a common effect observed with high concentrations of all exogenous auxin treatments examined (Figure 4e,j,o). Unlike 2,4-D and NAA, 10 μM IAA was unable to promote rhizoid development along the margins of the prothallus (Figure 4o), illustrating the variation in plant response to auxin compounds. For this reason, we continued to use all three auxin chemicals for subsequent analyses.

KYN is a selective inhibitor of auxin biosynthesis which is known to act by serving as an alternative substrate to the TAA1 enzyme in Arabidopsis root tissue at concentrations as low as 30 μM [20]. We observed that Ceratopteris gametophytes had greater resistance to KYN treatment, as 100 μM KYN was unable to decrease the cell number (907 cells ± 280) compared to the solvent mock (1004 cells ± 239) (Figure 4c’). At this concentration, KYN only appeared to reduce the width of the prothallus to create a more circular appearance, while rhizoids and gametangia developed normally (Figure 4q,s). 200 μM KYN was sufficient to induce morphological changes in the gametophytes, which appeared much smaller and circular than the corresponding solvent mock (Figure 4r,t). These gametophytes had shorter rhizoids and produced significantly fewer cells (732 cells ± 176) compared to the respective solvent mock (1520 cells ± 257) (Figure 4c’), although meristem placement was unaffected.

NPA is a polar auxin transport inhibitor first identified to have severe effects on plant morphology by Hoffmann and Smith [21]. More recently, NPA was characterized to directly bind PIN transporters at a unique site from IAA and interfere with PIN dimerization [22]. Ceratopteris gametophytes grown on 50 μM NPA maintained a chordate shape and proper gametangia formation, with fewer but more elongated cells (397 cells ± 97) compared with the corresponding solvent mock (556 cells ± 108) (Figure 4v,x,d’). While gametophytes grown on 100 μM NPA had significantly fewer cells (171 cells ± 47) compared to basal media-grown gametophytes (955 cells ± 271), an equal amount of 95% ethanol used in the solvent mock resulted in a similar decrease in cell number (256 cells ± 79) (Figure 4u,w,y,d’). Therefore, for later experiments, we switched the solvent to DMSO to avoid further off-target effects of the solvent concentration in the media.

### 2.5. Auxin Affects the Marginal Meristem Position

**Figure 5:**
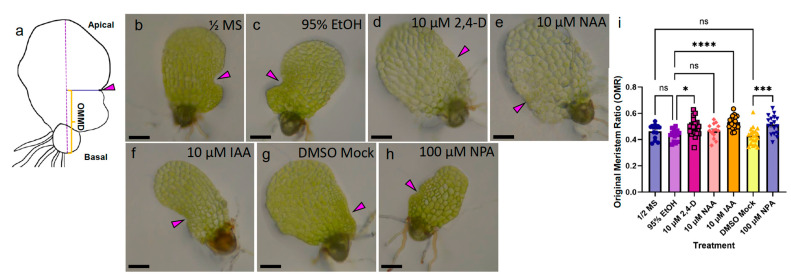
Exposure to high doses of NPA and IAA during marginal meristem formation alters hermaphrodite meristem location. (**a**) Diagram of original marginal meristem distance (OMMD) measurement scheme. Long-dashed line marks the apical–basal axis of the prothallus. Solid blue line marks the location of original meristem (magenta arrowhead), perpendicular to the apical–basal line. Orange bracket marks the OMMD. (**b**–**h**) Bright field micrographs of live 6-dpp hermaphrodites on treatment media: (**b**) Basal ½ MS media control. (**c**) 95% EtOH solvent mock for 2,4-D, NAA, and IAA, (**d**) 10 μM 2,4-D, (**e**) 10 μM NAA, (**f**) 10 μM IAA, (**g**) DMSO solvent mock for NPA, (**h**) 100 μM NPA. (**i**) OMR for treatments shown in (**b**–**h**) (*n* ≥ 14, mean ± SEM, Kruskal–Wallis with Dunn’s multiple comparisons test; ns, not significant; *, *p* ≤ 0.05; ***, *p* ≤ 0.001 ****, *p* ≤ 0.0001). Scale bars = 0.1 mm.

Cell proliferation transitions from the apical meristem to the marginal meristem over the course of 5- to 7-dpp of gametophyte development. To discern the specific action of auxin chemicals on the marginal meristem position, we transferred 4-dpp hermaphrodites onto media containing the highest of our tested concentrations for two days: 10 μM 2,4-D, 10 μM NAA, 10 μM IAA, and 100 μM NPA (KYN was omitted because it did not affect meristem morphology in the previous assay) (Figure 5a–i). After treatment, the original marginal meristem distance (OMMD) was measured from the position of the marginal initials to the basal end of the prothallus at the midline (Figure 5a). The marginal meristem ratio (OMR; see Methods for details) describes the meristem position while minimizing variation in the gametophyte length caused by NPA, NAA, and 2,4-D treatment (Figure S5). 10 μM 2,4-D, 10 μM IAA, and 100 μM NPA caused a significant increase in OMR compared to the corresponding solvent mocks (Figure 5i). The disruption of auxin gradients during marginal meristem formation by the limitation of polar auxin transport or exogenous application of auxins 2,4-D and IAA, but not the synthetic auxin NAA, are sufficient to shift the position of the marginal meristem toward the apical end of the gametophyte. The capability of 2,4-D, IAA, and the transporter inhibitor NPA to alter the position of the marginal meristem suggests that the proper transport of auxin and local concentrations across the prothallus play a role in meristem location.

### 2.6. Disruption of Auxin Homeostasis Affects Meristem Regeneration after Ablation

In the liverwort *M. polymorpha*, a transient decrease in auxin after excision of the apical meristem triggers regeneration in the meristem-less basal region, a process inhibited by the application of exogenous NAA [17]. In our system, the disappearance of and the appearance of *CrTAA2* expression at the sites of ablation and regeneration, respectively (Figure 3i,j), suggests that the destruction of a single marginal initial cell is sufficient to abolish auxin biosynthesis at that site. This alteration of *CrTAA2* expression led us to hypothesize that the local auxin optimum may play a role in meristem regeneration. To test this, we disrupted the change of the local auxin concentration using auxin chemicals.

**Figure 6:**
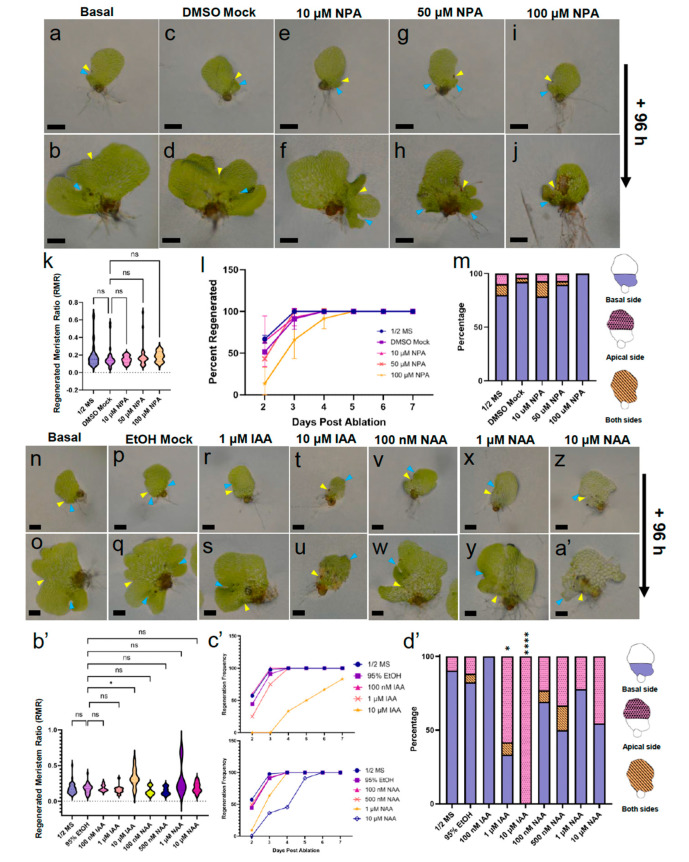
NPA, NAA, and IAA dosage during recovery from laser ablation causes abnormalities in marginal meristem regeneration. (**a**–**j**,**n**–**a’**) Live gametophytes on treatment media 2- or 3-days post-ablation and the same individuals 96 h later. (**a**–**m**) Effects of NPA dosage during meristem regeneration. (**n**–**d’**) Effects of NAA and IAA dosage during meristem regeneration. (**k**,**b’**) Violin plot of regenerated meristem ratio (RMR) values for (**k**) NPA dosage (*n* ≥ 22) and (**b’**) NAA or IAA dosage (*n* ≥ 9) (dashed line = median; dotted lines = upper and lower quartiles; Kruskal–Wallis test with Dunn’s multiple comparisons test; ns, not significant; *, *p* ≤ 0.05). (**l**,**c’**) Percentage of regenerated individuals from 2- to 7-days post-ablation during treatment with (**l**) NPA or (**c’**) NAA and IAA. (**m**,**d’**) Percentage of individuals with a regenerated meristem in the basal (solid), apical (dotted), or both (striped) portions of the prothallus (*n* ≥ 9, Fisher’s exact test; *, *p* < 0.05; ****, *p* < 0.0001). Yellow and blue arrowhead pointing at ablated and regenerated meristem, respectively. Scale bar = 0.25 mm.

**Figure 7:**
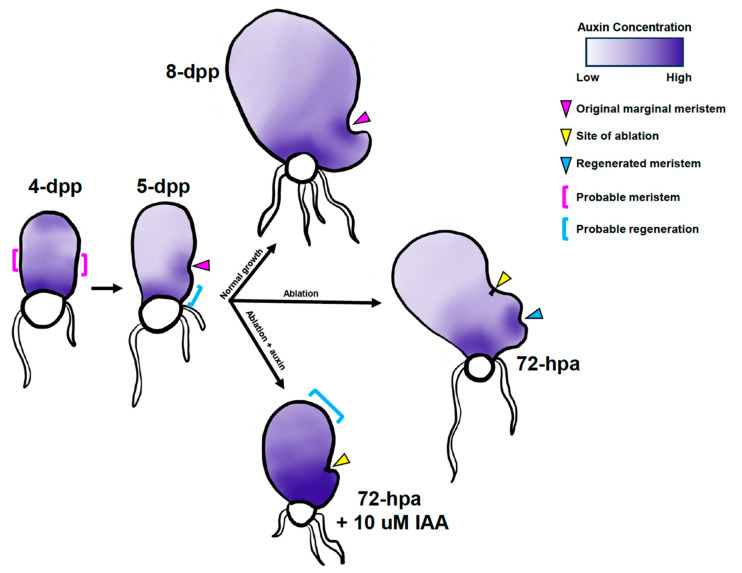
Model of auxin influence on marginal-meristem formation and regeneration. Hypothetical auxin gradients are represented by intensity of indigo color. 4-dpp gametophytes have not specified a marginal meristem and have an intermediate concentration of auxin between the apex and base of the prothallus, optimum for marginal meristem formation. 5-dpp gametophytes select one side to specify the marginal meristem, which becomes a site of auxin biosynthesis. The region between the original meristem and basal end has an intermediate concentration of auxin, which is optimum for regeneration. 8-dpp gametophytes experiencing normal growth continue to exhibit a similar auxin gradient pattern as the 5-dpp ones. Gametophytes ablated at 5-dpp regenerate a new meristem in the region with optimum auxin levels, most frequently between the ablation site and basal end. Gametophytes ablated and treated with 10 μM IAA experience a higher concentration of IAA throughout the gametophyte, which raises the auxin levels in the basal end beyond the level which promotes regeneration. Therefore, the apex of the gametophyte becomes optimum for regeneration after some delay.

NPA significantly reduced cell proliferation of the non-ablated gametophytes after 14 days of treatment (Figure 4w–x,c’) and increased the OMR in those treated with NPA from 4- to 6-dpp (Figure 5g,h). Therefore, we chose to test the effect of NPA on the regeneration process post-ablation. 5-dpp gametophytes were ablated and placed on basal (Figure 6a,b), DMSO solvent mock (Figure 6c,d), 10 μM (Figure 6e,f), 50 μM (Figure 6g,h), or 100 μM NPA (Figure 7i,j) media for seven days. At 48-hpa, the RMD values were calculated as described in Figure 2. Due to the effect of NPA on the overall gametophyte cell number (Figure 4c’) and size (Figure S6a–c), the location of the new meristem was expressed as a ratio of meristem distance per μm gametophyte length, which we called the regenerated meristem ratio (RMR) (see Figure S6d for raw distance values). 10 μM NPA-, 50 μM NPA-, and 100 μM NPA-treated gametophytes exhibited a median RMR comparable to the DMSO solvent mock, although the 100 μM NPA-treated group was trending toward significance (*p* = 0.15) in comparison to either of the lower concentrations of NPA (*p* > 0.99) (Figure 6k). The percentage of individuals with visible regeneration was scored from 2- to 7-days post-ablation during NPA treatment. Like the DMSO solvent mock, 10 μM and 50 μM NPA-treated gametophytes had reached 100% regeneration frequency by the fourth day of recovery, while the 100 μM NPA gametophytes experienced a developmental delay of one day (Figure 6l). NPA did not have a significant effect on the regeneration position within the apical or basal portion of the prothallus (Figure 6m). The concentrations of NPA tested were unable to promote meristem regeneration farther from the site of ablation (Figure 6k), unlike the effect of NPA on the original meristem position. This suggests that polar auxin transport is involved in determining the location of the original meristem but has less of an effect on the regenerated meristem position.

We repeated our analyses using treatments with 2,4-D (100 nM, 1 μM, and 10 μM) and KYN (100 μM and 200 μM) (Figure S7a–j,n–u). The 2,4-D treatment was able to delay regeneration, as the 10 μM 2,4-D-treated gametophytes took an additional two days to reach 100% regeneration compared to the solvent mock group (Figure S7l). KYN had a weak dosage-dependent inhibitory effect on regeneration after two days, but all groups were able to reach 100% regeneration by four days of treatment post-ablation (Figure S7w). The RMR values were unaffected by treatment with different concentrations of 2,4-D or KYN (Figure S7k,v, Figure S8a–c,e–g; see raw distance values in Figure S8d,h). Likewise, the position of the regenerated meristem was preferentially on the basal side of the prothallus for the basal media controls, solvent mocks, and all treatments of 2,4-D and KYN examined here (Figure S7m,x). The results of our KYN experiments show that inhibition of auxin biosynthesis after ablation is not sufficient to alter the positioning of the regenerated meristem, only to delay cell proliferation (Figure S7n–x). This suggests that existing local auxin gradients at the time of ablation guide the positioning of meristem regeneration.

Next, we decided to treat ablated gametophytes with NAA or IAA during the ablation recovery period. A range of concentrations (NAA: 100 nM, 500 nM, 1 μM, and 10 μM; IAA: 100 nM, 1 μM, and 10 μM) was used to dose the ablated gametophytes, followed by an evaluation of the regeneration (Figure 6n–a’). RMR was again used to determine the meristem location (Figure S6e–g; see Figure S6h for raw distance values). The RMR values of the 100 nM IAA, 1 μM IAA, and all NAA-treated samples did not differ significantly from the ethanol solvent mock (Figure 6b’). A Notable effect on the RMR were observed exclusively in the 10 μM IAA-treated gametophytes, which was significantly higher than the solvent mock (Figure 6b’). Of the treatments used, 1 μM IAA and 1 μM NAA were the lowest concentrations tested able to delay regeneration, with 25% (1 μM IAA) or 9% (1 μM NAA) of the treated individuals exhibiting visible regeneration, compared to 52% of the solvent mock-treated gametophytes after 2 days (Figure 6c’). 10 μM NAA further inhibited regeneration, with 0% of the gametophytes regenerated after 2 days and 100% at 6 days, a full two days later than the solvent mock (Figure 6c’). 10 μM IAA had the most pronounced effect on regeneration, with 0% of the ablated gametophytes regenerating after 3 days of recovery (Figure 6c’). After 7 days, only 83% of the 10 μM IAA-treated gametophytes had regenerated; the 10 μM IAA treatment was the only condition tested that successfully prevented regeneration past day 5 of treatment (Figure 6c’). The regenerated meristems exposed to 10 μM IAA lacked the hallmarks of meristematic activity, such as increased cell density and archegonium formation. Instead, these meristems had enlarged cells indistinguishable from other prothallium cells (Figure 6t–u). In contrast, the 10 μM NAA-treated gametophytes still regenerated meristems with typical small cells at a “notch” at the margin (Figure 6z–a’). Interestingly, the 1 μM and 10 μM IAA-treated gametophytes had significantly more regenerated meristems on the apical side (58% and 100%, respectively) compared to the solvent mock (12%) (Figure 6d’). Taken together, these results demonstrate that exogenous auxin is capable of delaying cell proliferation at the regenerated meristem. Perturbations of the level of naturally occurring auxin by exogenous IAA treatment produces a distinct effect on the distance of regeneration from the site of damage and the position of the meristem in the basal or apical half of the prothallus.

## 3. Discussion

The marginal meristem of a Ceratopteris hermaphroditic gametophyte has been described to have a single apical “initial cell” [8] or three marginal initials [5]. The behavior of the non-ablated and ablated gametophytes found in our study provides support for the two conflicting descriptions. Our in situ hybridization data of *CrTAA2* and *CrWOXB* expression in the 5-dpp gametophytes showed localized expression in a wide area of the marginal meristem; however, from 7-dpp onward, a few cells at the center of the meristem distinguished themselves by a decreased hybridization signal of these two genes (Figure 3). The expression pattern of these two genes suggests a transcriptional shift in these cells that may be responsible for the increased proliferative potential of the marginal initials. These cells are in comparable positions as those marginal initials with high proliferative activity described by Geng et al. [5].

The three marginal initials of the meristem described in Geng et al. [5] contribute similarly to a gametophyte’s cell number, so it would seem that the loss of one marginal initial would reduce the amount of proliferation without disruption of the meristem structure. However, we did not observe this for gametophytes with an ablated marginal initial (Figure 1). Our single-cell ablation system revealed the importance of a single marginal initial on sustained meristematic activity, as ablation of only one cell was enough to prohibit further divisions at the original location and trigger the regeneration of a marginal meristem at a new location (Figure 1 and Figure 2), a result similar to the ablation of the AC-based meristem in *T. palustrus* [12]. Importantly, damage alone was not enough to initiate regeneration (Figure S1); specific damage to the meristem was necessary to induce regeneration. This is in agreement with the observation that damaging a group of cells outside of the meristem has no effect on new meristem regeneration [5]. Our results indicate that the death of one marginal initial cell is sufficient to disrupt the meristem; therefore, each of the marginal initial cells may play specific roles to organize the marginal meristem structure and/or inhibit ectopic meristem formation during development. The quick transfer of propidium iodide between the targeted marginal initial cell and its neighboring initial cell post-ablation (Figure 1a) implies increased connectivity between the initial cells via plasmodesmata, a phenomenon suggested by Bartz and Gola [8]. It remains a future area of research to discover the signaling molecules associated with initiating and maintaining the identity of the marginal initial cells in the fern gametophyte.

While the new meristem regenerated after ablation appears morphologically similar to a non-ablated 7-dpp marginal meristem (Figure 1c), we were surprised by the lack of CrWOXB expression in the regenerated meristem (Figure 3). It is possible that CrWOXB is expressed before or after the time sampled during our analysis. Another possibility is that a different CrWOX gene is responsible for maintaining proliferation at this new meristem, such as auxin-responsive CrWOXA or marginal meristem-expressed CrWUL [23,24,25].

The shape of the gametophyte and the meristem morphology of *Ceratopteris* species are sensitive to treatment of exogenous auxins, such as 2,4,5-T and NAA in *C. richardii* [2] or 2,4-D, NAA, and IAA in *C. thalictroides* [26]. We observed similar changes during our experiments with 2,4-D, NAA, and IAA (Figure 4), including the formation of a marginal meristem at the apical end as opposed to the lateral region. Taking into consideration these variations in growth and shape, we performed a detailed analysis to understand the marginal meristem position. NPA, 2,4-D, and IAA were able to cause gametophytes to form the marginal meristem farther away from the basal end of the gametophyte (Figure 4 and Figure 5) after only two days of treatment (Figure 4 and Figure 5). The lack of polar auxin transport in the NPA-treated gametophytes mimicked exogenous auxin treatment (Figure 5), indicating that changes in local auxin maxima may be responsible for determining the site of the marginal meristem. In contrast, the ablated gametophytes treated with NPA did not form new meristems farther away from the site of ablation(Figure 6). We observed that *CrTAA2* expression disappeared from the site of ablation (Figure 3), suggesting a flattening or disappearing of an auxin gradient from the ablated meristem. To observe the effect of polar auxin transport during regeneration, a higher concentration of NPA treatment may be necessary. An alternative possibility is that polar auxin transport does not influence the position of the regenerated meristem.

In other wound regeneration systems, such as callus formation in Arabidopsis and thallus regeneration in liverwort, treatment with synthetic auxin inhibits these processes [17,27]. Meristem regeneration in Ceratopteris gametophytes responded to auxin differently; treatments with 2,4-D and NAA were able to delay but were unable to prevent the gametophytes from specifying a new meristem, and eventual regeneration occurred within the 7-day recovery period after ablation (Figure 6). The naturally occurring IAA was the most effective, as 10 μM IAA was able to partially inhibit regeneration seven days post-ablation (Figure 6). The regenerated meristems showed an atypical morphology; the area of proliferation did not contain signature anticlinal divisions and increased cell density seen in marginal meristems and instead appeared distorted with enlarged cells (Figure 6). Interestingly, IAA treatment biased regeneration toward the apical side of ablation (Figure 6), similar to the behavior of the non-ablated gametophytes germinated on IAA (Figure 4). An increased IAA concentration across the prothallus appeared to promote an apical meristem location, implying that during normal development, the apical end does not have a sufficiently high concentration of auxin for a marginal meristem to form.

The ability to regenerate a new meristem after ablation and relocation of the regeneration sites by auxin illustrate the capacity of prothallus cells to dedifferentiate and acquire a marginal initial identity, an ability which is inhibited by an existing meristem elsewhere, likely through some form of gradient that involves auxin. We chose to ablate the marginal initial cells soon after the acquisition of the marginal meristem. It remains to be explored if regeneration efficiency is reduced in gametophytes further developed (such as 7- to 10-dpp hermaphrodites).

A model of how auxin gradients affect marginal meristem formation and regeneration is presented (Figure 7). During specification of the original marginal meristem, auxin produced by the dividing cells in the apical domain is transported toward the basal end near the rhizoids and accumulates there. Auxin is known to induce rhizoid production in *C. thalictroides* [26], liverworts, and *Physcomitrium patens* (reviewed in Jones and Dolan [28]), as we have observed in *C. richardii* (Figure 4). This gradient of auxin creates an optimal concentration for marginal meristem specification and proliferation on either the left or right margin in the middle of the prothallus. Established meristems synthesize auxin via CrTAA2, which together with polar auxin transport, forms a new gradient away from the marginal initials. Upon ablation, auxin synthesis stops, triggering the regeneration of a new meristem where auxin is at the optimum concentration. Most often, the new meristem is formed at the basal side (where the newer lobe is), as it is adjacent to the auxin sink. If auxin levels are altered by treatment with IAA, the optimal concentration becomes closer to the apical end, which under normal conditions would not have enough auxin to promote cell proliferation and instead induce cell elongation. Future analyses with auxin-sensitive reporters will provide critical support for the model.

In conclusion, our method of single-cell laser ablation of marginal initial cells has clarified the definition of “initial cells” of the marginal meristem in Ceratopteris gametophytes. Identifying a *TAA1* homolog, *CrTAA2*, allowed us to discover a bilateral (on both sides of the initial cells) expression pattern similar to the known cell-proliferation marker *CrWOXB* at the original meristem and the re-establishment of *CrTAA2* expression at the regenerated meristem. Lastly, by a quantitative analysis of gametophyte cell number and shape dynamics, we uncovered a role for auxin in determining the meristem location in normal gametophyte development and in response to the loss of a marginal initial cell to ablation.

## 4. Materials and Methods

### 4.1. Plant Materials and Growth Conditions

*Ceratopteris richardii* spores of the strain RN3 (Carolina Biological Supply Company, Burlington, NC, USA) were used for all experiments and sterilized as described in Withers et al. [29]. RN3 spores were briefly surface sterilized with a solution of 4% sodium hypochlorite and 0.1% Tween-20 for 3 min, rinsed five times with sterile water, and dark-treated for five days to synchronize germination. Spores were plated on basal media (1/2 strength Murashige & Skoog salts, Phytotech Labs, Lenexa, KS, USA; pH 6.0, 0.8% agar, 100 mg/L ampicillin) and grown at 26 °C under humidity domes under the lighting conditions described by Youngstrom et al. [25] for the duration of growth, hormone treatment, and recovery after laser ablation.

### 4.2. Laser Ablation of Marginal Meristem Initials

At the time of marginal meristem specification (5- to 6-days post-plating spores (dpp)), individual hermaphroditic gametophytes were selected. For each gametophyte, the central cell of the three marginal meristem initials was identified based on morphology, according to Bartz and Gola [8], and targeted at 40× magnification on a Zeiss Axio Observer A1 inverted microscope (Oberkochen, Germany) with a Micropoint Laser Illumination and Ablation System (Andor Technology: Oxford Instruments, Abingdon, UK) and exposed to the laser beam for 3 s to ablate the cell within the plane of focus. Immediately after ablation, gametophytes were placed on agar treatment media or recovery media. For verification of laser ablation, gametophytes were stained with a 10 μM aqueous solution of propidium iodide and fluorescence signal visualized before and after ablation with a Zeiss filter set 20 (excitation: BP 546/12; emission: BP 575–640) of the inverted microscope described above and an attached Andor Zyla sCMOs camera. Greyscale images were pseudo-colored according to pixel intensity values with a magenta-orange color scheme. Corresponding greyscale bright field images were acquired with the same technique.

### 4.3. Phylogeny of Ceratopteris TAA Proteins

Sequence data were acquired from NCBI [30], Phytozome 13 [31], Fernbase [32], and TAIR [33] (specific sequence ID numbers and genome databases are listed in Table S1). The AtTAA1 (AT1G70560) amino acid sequence was used as the BLASTp query on Phytozome to identify homologs in Ceratopteris. The sequences identified were aligned in Jalview 2.11.2.7 [34] using Clustal Omega (ClustalO, version 1.2.4) with default parameters (transition matrix: Gonnet; gap opening penalty: 6 bits; gap extension: 1 bit). The Ceratopteris sequence with the highest percent identity to AtTAA1, CrTAA1 (Ceric. 10G039800), was then used as the query for BLASTp searches on Fernbase to identify potential homologs in *Azolla filiculoides*, *Salvinia cucullata*, *Adiantum capillus-veneris*, *Alsophila spinulosa*, and *Marsilea vestita*. Protein sequences from other angiosperms and bryophytes were acquired by searching Phytozome for existing tryptophan aminotransferase KEGG annotations and by using CrTAA1 as a BLASTp query. Algae genomes on NCBI were also queried in the same manner. A total of 61 amino acid sequences were aligned with ClustalO with the same parameters described above, which was used to construct a maximum likelihood tree in MEGAX [35] using the JTT model substitution method and 500 bootstrap replicates.

### 4.4. Real-Time Quantitative PCR and Whole Mount In Situ Expression Analyses

50 mg tissue samples of a mixed population of males and hermaphrodites of 7, 10, and 13 dpp gametophytes were harvested for RNA extraction and flash-frozen in liquid nitrogen and stored at −75 °C. Total RNA was extracted using the Quick-RNA MiniPrep (Plus) kit (Zymo Research, Irvine, CA, USA) and used for cDNA synthesis, as described previously [25,29]. Expression of *CrTAA1*, *CrTAA2*, *CrTAA3,* and *CrYUC2-3* were measured relative to *CrUBQ* using a QuantStudio™ 3 Real-Time PCR System (Applied Biosystems, ThermoFisher Scientific, Waltham, MA, USA), as described in Withers et al. [29]. Three biological and two technical replicates were performed for each time point. The fold change expression was calculated using the ΔΔCt method [36].

Templates for probe synthesis of the CrTAA2 and CrYUC2-3 sequences were cloned into the pENTR/D-TOPO vector (Life Technologies, Carlsbad, CA, USA). Antisense and sense RNA in situ probes were synthesized, as described previously [14]. The primer sequences for RT-qPCR, cloning, and RNA probe templates are listed in Table S2. Tissues used for whole mount in situ hybridization (5-, 7-, and 9-dpp non-ablated hermaphrodites and 72-h post-ablation (hpa) hermaphrodites) were harvested from agar media and fixed in FAA (formaldehyde: ethanol: acetic acid; 3.7%:50%:5% *v*/*v*, respectively) at room temperature for 2 h. Fixative was replaced with 70% ethanol and stored at −20 °C until use. Whole mount in situ hybridization was performed based on Hejatko et al. [37], starting from the permeabilization of samples with 1:1 ethanol: Histoclear II with the following modifications: the hybridization solution contained 150 μg mL^−1^ of yeast tRNA and either 1 ng uL^−1^ (*CrTAA2*) or 0.5 ng uL^−1^ (*CrWOXB*, *CrYUC2-3*) of the DIG-labeled RNA probe, the pre-absorption of diluted Anti-Digoxigenin-AP Fab fragments was omitted, the antibody incubation time was increased to 20 h, and samples underwent color development staining for 5 h with 500 μM levamisole. Stained tissues were mounted in 50% glycerol and imaged on a Zeiss compound light microscope with a Zeiss Axiocam Erc 5s digital camera (Carl Zeiss Microscopy, LLC, Thornwood, NY, USA).

### 4.5. Exogenous Auxin and Auxin Inhibitor Treatments of Gametophytes

Gametophytes were treated on basal media containing synthetic auxins 2,4-dichlorophenoxyacetic acid (2,4-D; Sigma-Aldrich, St. Louis, MO, USA) or 1-naphthaleneacetic acid (NAA; Sigma-Aldrich), naturally occurring auxin indole-3-acetic acid (IAA; Sigma-Aldrich), auxin biosynthesis inhibitor L-kynurenine (KYN; Sigma-Aldrich), or polar auxin transport inhibitor naphthylphthalamic acid (NPA; PhytoTech Labs, Lenexa, KS, USA). For treatments of the non-ablated gametophytes germinated on auxin chemicals, the following solvents were used to dissolve each compound: 1 M KOH, 2,4-D; 95% ethanol, 2,4-D, NAA, IAA, NPA; DMSO, NPA, KYN. For the assays of auxin-treatment effects on the original marginal meristem formation and meristem regeneration of the ablated gametophytes, plants were germinated on basal media and transferred to auxin media for the specified treatment length, 2 and 7 days, respectively. Gametophytes were imaged on media plates through the ocular lens of an Olympus compound microscope using a Nikon D3200 DSLR camera.

### 4.6. Quantification of Auxin Treatment Response on Non-Ablated and Ablated Hermaphrodites

Gametophytes germinated on auxin chemicals were harvested at 11-dpp (2,4-D, NAA, and IAA), 12-dpp (KYN), or 14-dpp (NPA) and cleared in 95% ethanol at 4 °C for at least 24 h, followed by staining with 40 μg mL^−1^ Hoechst 33342. Stained gametophytes were mounted in 50% glycerol, then imaged and quantified, as previously described [25,29]. Images of live gametophytes were measured in FIJI using the Straight Line Tool (length, width, distance) or the Freehand Selection Tool (area). “Length” was defined as the distance from the basal (spore) to apical side, bisecting the gametophyte body, with “width” drawn perpendicular to the length line across the widest part of the gametophyte. To determine the position of the original marginal meristem, a line from the meristem center was drawn perpendicular to the length line until the two lines intersected. The distance from the intersection point to the base of the gametophyte was defined as the original marginal meristem distance (OMMD). The original meristem ratio (OMR) was calculated by dividing the OMMD by the total μm gametophyte length. The regenerated meristem distance (RMD) represented the distance between the site of ablation and the center of the regenerated marginal meristem, which we used to calculate the regenerated meristem ratio (RMR): RMD divided by total μm of the gametophyte length. The regeneration frequency over time was quantified by binary scoring images of ablated gametophytes from 2- to 7 days for the presence or absence of visible regeneration (anticlinal divisions, increased cell density, invagination of margin, and archegonium production) and was reported as a percentage of the total sample size. Gametophytes were classified into three groups based on the directionality of regeneration (basal, apical, or both) with respect to the site of ablation and were reported as percentage of the total.

### 4.7. Statistical Analyses and Graphical Representations

All statistical analyses and chart productions were performed in GraphPad Prism 10.0.2 [38]. Comparisons of cell counts of the auxin-dosed gametophytes were calculated using either a Kruskal–Wallis with Dunn’s post-hoc test (2,4-D and NPA), a one-way ANOVA with Sidak’s post-hoc test (KYN and NAA), or a Dunnett’s post-hoc test (IAA). Measurements of the auxin-dosed marginal meristem position gametophytes’ length, width, and area were conducted using a one-way ANOVA with Šídák’s multiple comparisons test. Measurements of the ablated gametophytes’ length, width, and area were compared with a Kruskal–Wallis with Dunn’s comparisons test (KYN—length and area; IAA/NAA—length, width, and area; NPA—length and width), a one-way ANOVA with Šídák’s multiple comparisons test (2,4-D—length, width, and area; KYN—width), or a Dunnett’s multiple comparisons test (NPA—area). Comparisons of the OMR and RMR values were determined using a Kruskal–Wallis with Dunn’s post-hoc test. For the RT-qPCR data, a two-way ANOVA with Tukey’s post-hoc test was used. Regeneration side comparisons of the observed count values were conducted by Fisher’s exact test with a Bonferroni correction of *p*-values. The basal media gametophyte heatmap of RMD was constructed using a representative gametophyte image with a demarcation of concentric rings centered on the site of ablation, from 50 to 400 μm in increments of 50 μm using the Circle tool in Fiji. This marked image was used as a template in Adobe Photoshop to create the heatmap, with each ring filled according to the frequency of individuals with RMD values in each 50 μm bin (minimum, 0%; maximum, 40%), mapped to a grid-lined color gradient marked in 5% intervals.

## Data Availability

Data available upon request.

## Supplementary Materials

The following supporting information can be downloaded at: https://www.mdpi.com/article/10.3390/ijms242115832/s1.
