# Supplementary material for: Auxin Involvement in Ceratopteris Gametophyte Meristem Regeneration
Source: Int J Mol Sci. 2023 Oct 31;24(21):15832. doi: 10.3390/ijms242115832 (PMC10647518; doi:10.3390/ijms242115832)
Supplement: Supplementary file 1 [file ijms-24-15832-s001.zip › Supplemental Figures with Captions Combined.pdf]

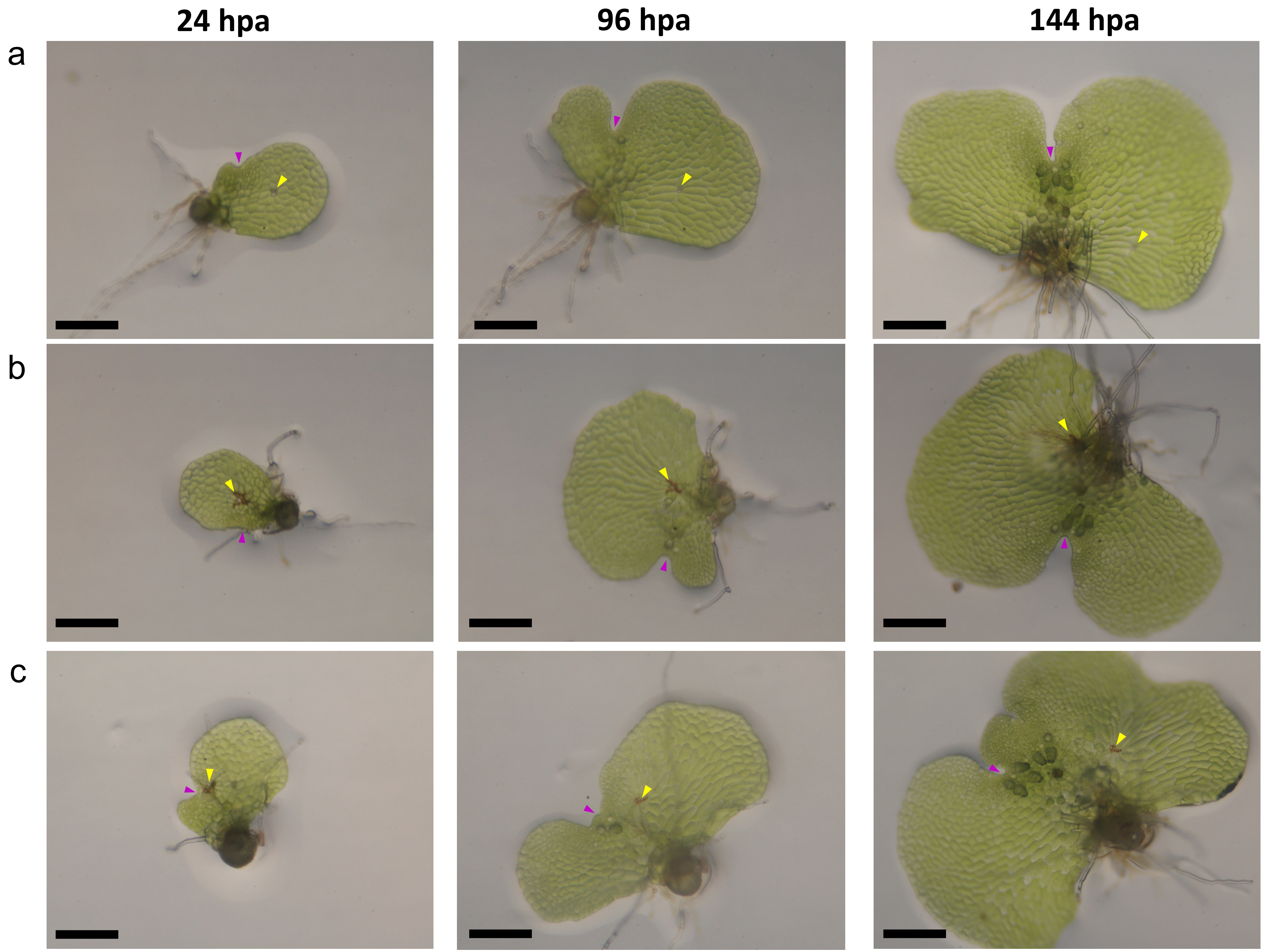

Supplemental Figure S1. Additional images of post-ablated damage sites. **(a–c)** Time course (24-, 96-, and 144-hpa) images of gametophytes that show no meristem regeneration with an ablated internal cell instead of the marginal initial cell. **(a)** Individual with central somatic cell ablated. **(b)** Individual with basal somatic cell ablated. **(c)** Individual with marginal initial adjacent cell ablated. Magenta and yellow arrowheads point at the meristem and site of ablation, respectively.

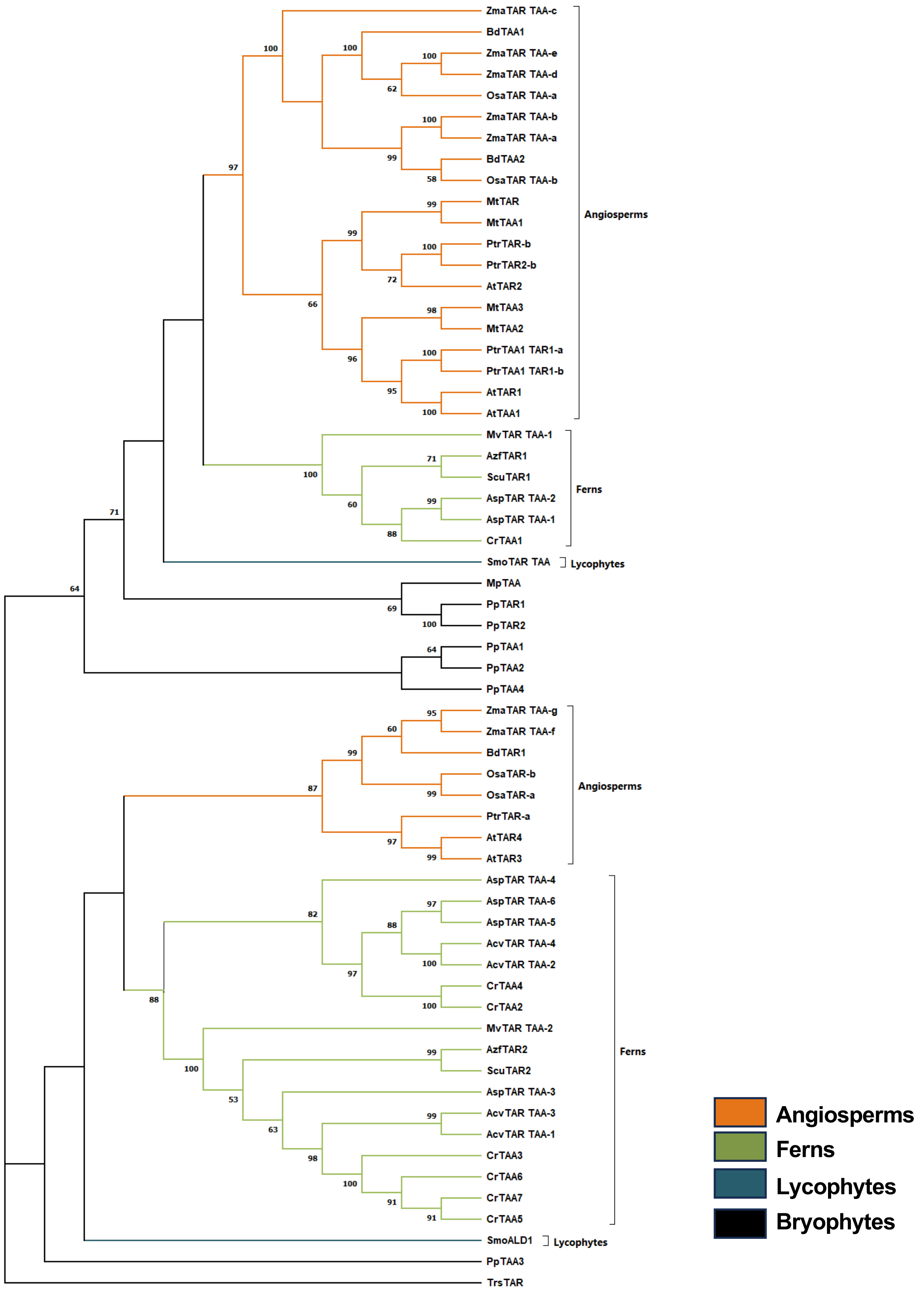

Supplemental Figure S2. Phylogenetic analysis of identified CrTAA genes. Maximum likelihood tree of tryptophan aminotransferase proteins from 17 representative species of land plants computed with 500 bootstraps by the JTT model substitution method. Subtrees containing proteins from ferns (green), angiosperms (orange), lycophytes (blue), and bryophytes (black) are marked. Bootstrap values below 50 are not shown. Abbreviations: *Arabidopsis thaliana* (At), *Ceratopteris richardii* (Cr), *Selaginella moellendorfii* (Smo), *Marchantia polymorpha* (Mp), *Physcomitrium patens* (Pp), *Oryza sativa* (Osa), *Brachypodium distachyon* (Bd), *Populus trichocarpa* (Ptr), *Medicago truncatula* (Mt), *Zea mays* (Zma), *Salvinia cucullata* (Scu), *Azolla filiculoides* (Azf), *Adiantum capillus-veneris* (Acv), *Alsophila spinulosa* (Asp), *Marsilea vestita* (Mv), and *Trebouxia sp* (Trs).

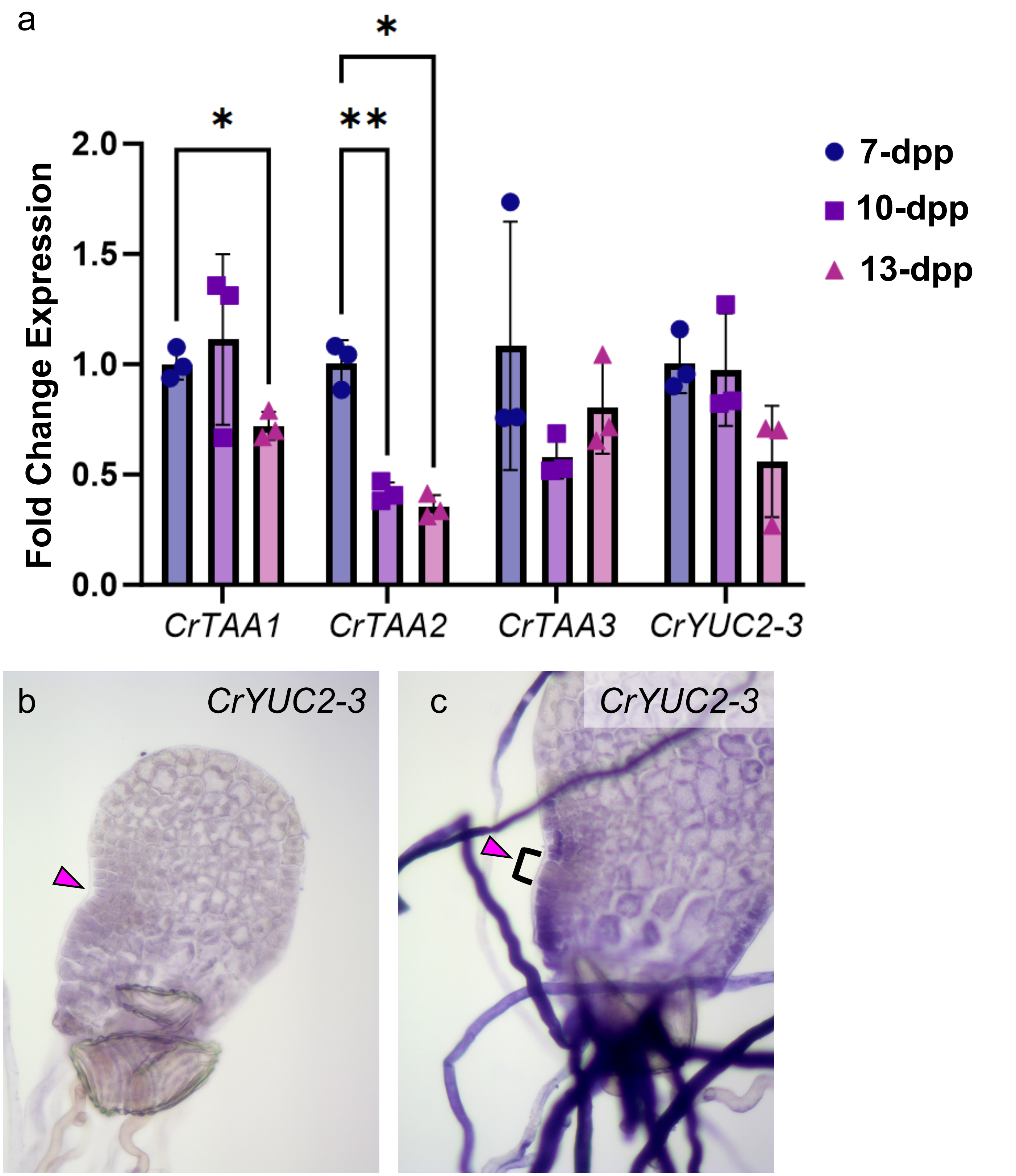

Supplemental Figure S3. RT-qPCR analysis of auxin synthesis genes during meristem development and localization of CrYUC2-3 (a) Bar graph of RT-qPCR fold change expression for *CrTAA1, CrTAA2, CrTAA3*, and *CrYUC2-3* in 7-, 10-, and 13-dpp gametophytes (Two-way ANOVA with Tukey's multiple comparisons test; *, $p < 0.05$; **, $p < 0.01$). (b,c) Whole mount in situ hybridization of *CrYUC2-3*: (b) sense probes and (c) antisense probes to 7-dpp gametophytes with visible meristems, indicated by magenta arrows. (c) *CrYUC2-3* expression present at the meristem, marginal initial cells marked with black bracket.

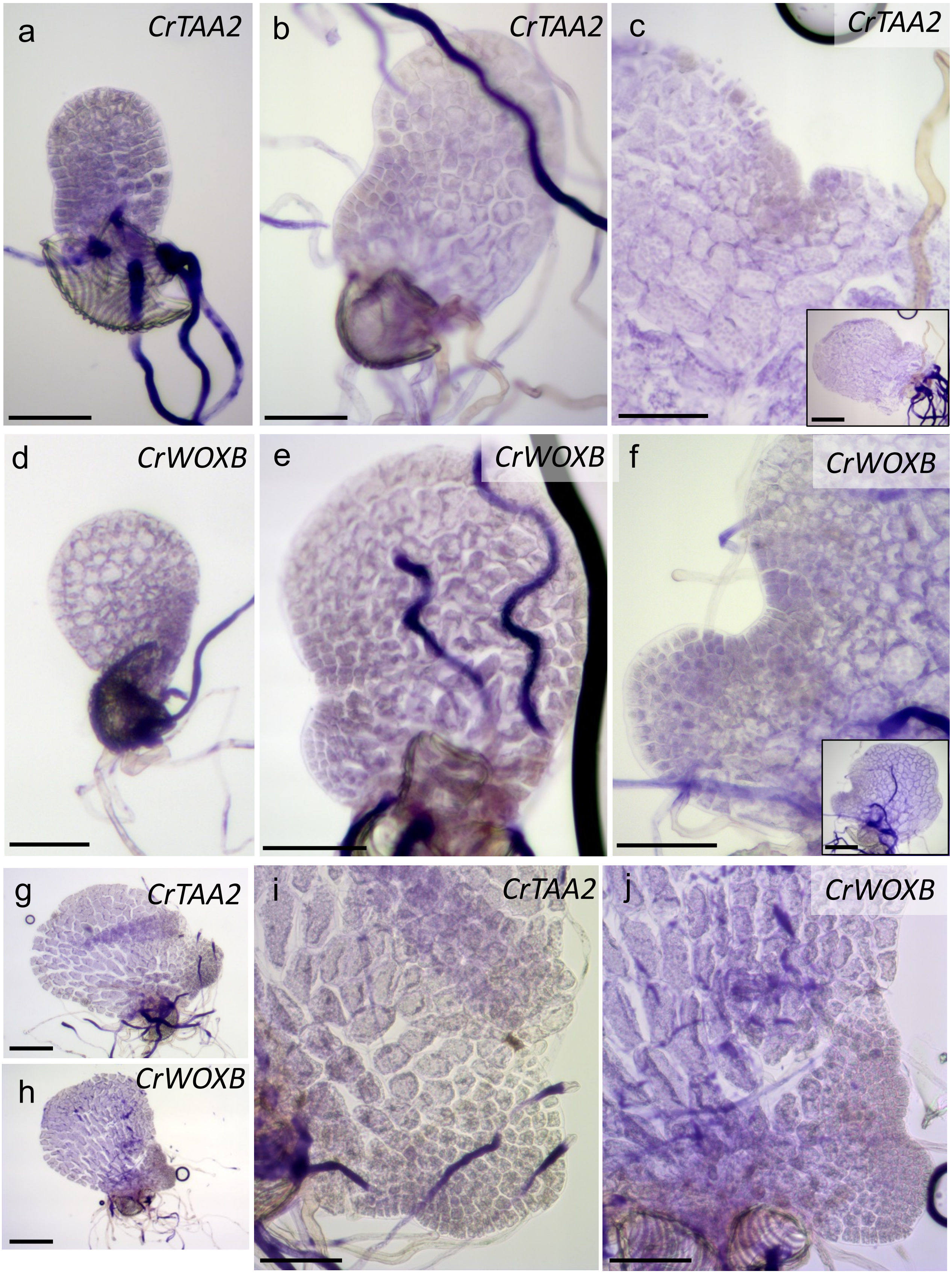

Supplemental Figure S4. *CrTAA2* and *CrWOXB* sense probe images for non-ablated and ablated gametophytes. (a–j) Whole mount in situ hybridization of (a–c,g,i) *CrTAA2* and (d–f,h,j) *CrWOXB* sense probes to gametophyte tissue: (a,d) 5-dpp hermaphrodites with a newly forming marginal meristem, (b,e) 7-dpp hermaphrodites with established marginal meristem and proliferating second lobe, (c,f) 9-dpp hermaphrodites with visible antheridia, continued cell proliferation in the marginal meristem, and beginning archegonia development. (g,h) 72-hpa hermaphrodites. (i,j) The site of ablation and regenerated meristems. (a–f,i,j). Scale bar = 0.2 mm, (c,f,g,h). Scale bar = 0.5 mm. (c,f) Inset scale bar = 0.5 mm.

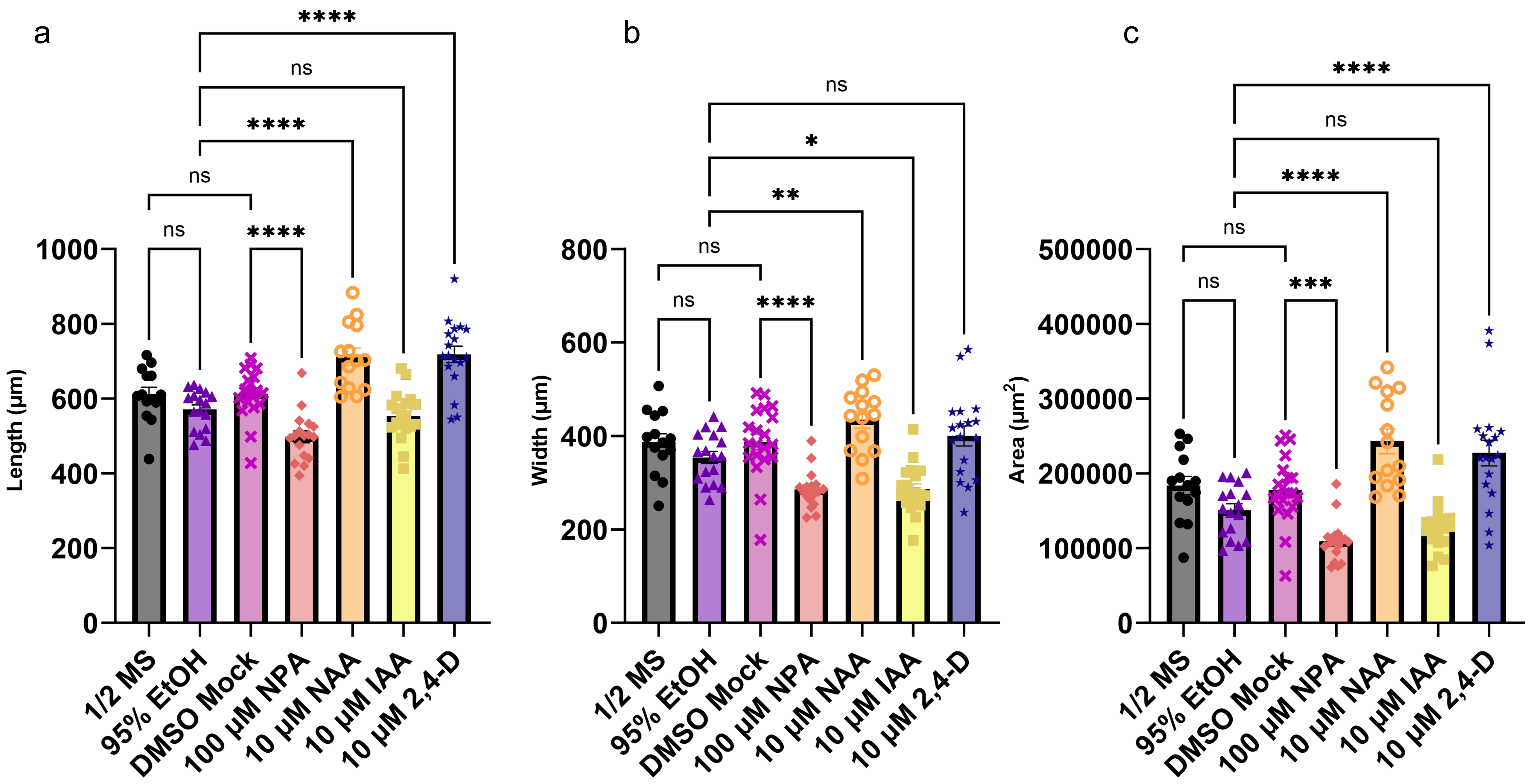

Supplemental Figure S5. Measurements of gametophyte shape during auxin chemical dosage from 4- to 6-dpp. (a–c) Gametophyte size and shape measurements collected for individuals used in OMR analysis ($n \geq 14$, mean □ SEM; one-way ANOVA with Sidak's multiple comparisons test; *, $p < 0.05$; **, $p < 0.01$; ***, $p < 0.001$, ****, $p < 0.0001$). (a) Length in µm from basal to apical end. (b) Width in µm perpendicular to the length axis. (c) Area in µm^2 of gametophytes.

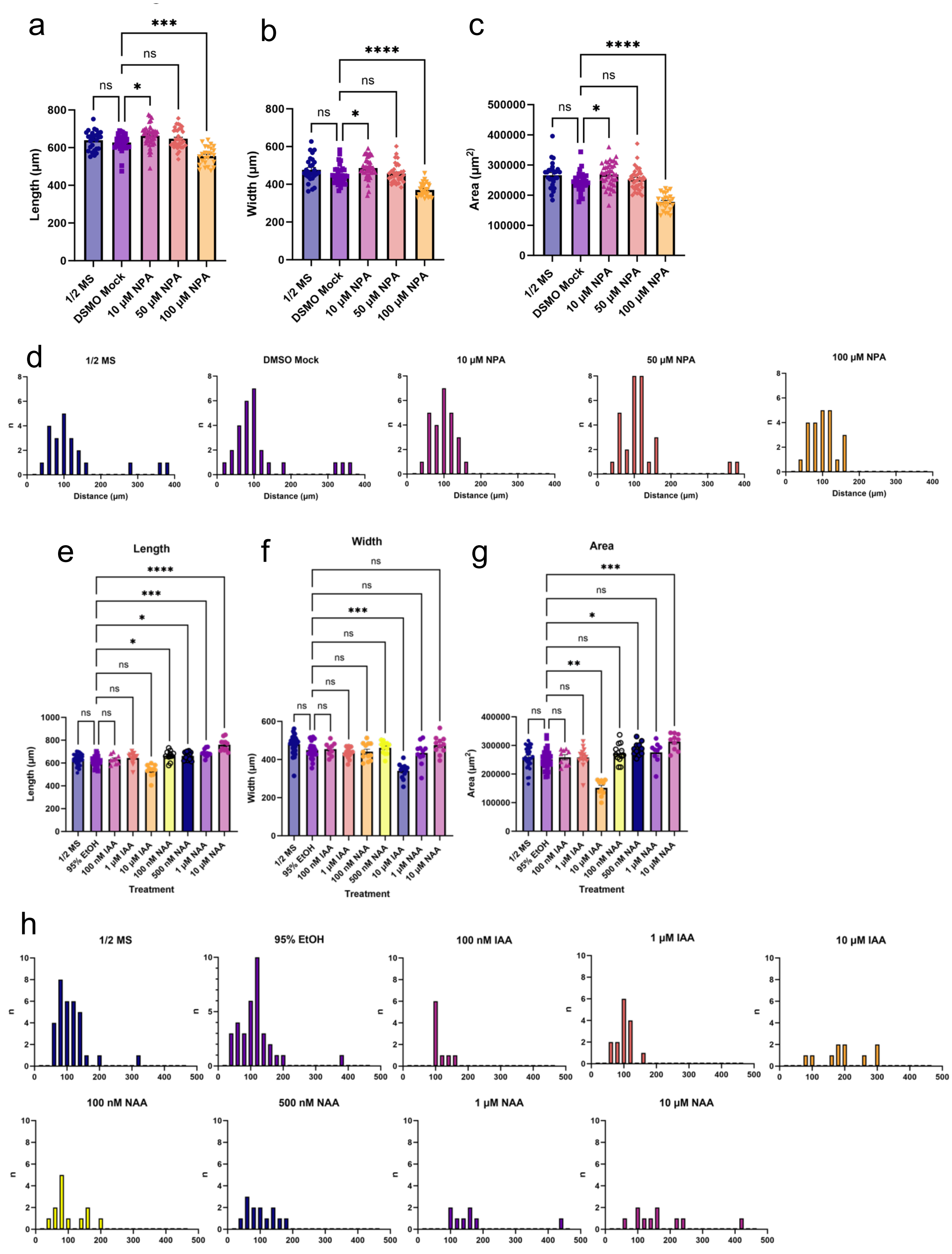

Supplemental Figure S6. Gametophyte shape and raw distance values for NPA, NAA, and IAA ablated dosage samples. (a–c,e–g) Gametophyte shape and size measurements for (a–c) NPA and (e–g) NAA/IAA treatments post-ablation. Length, width, and area in µm2 ($n \geq 22$, mean ± SEM; Kruskal–Wallis with Dunn's comparisons test (IAA/NAA—length, width, and area; NPA—length and width) or a one-way ANOVA with Dunnett's multiple comparisons test (NPA—area); *, $p < 0.05$; **, $p < 0.01$; ***, $p < 0.001$; ****, $p < 0.0001$). (d,h) Frequency histograms of raw RMD values for (d) NPA and (h) NAA or IAA treatment.

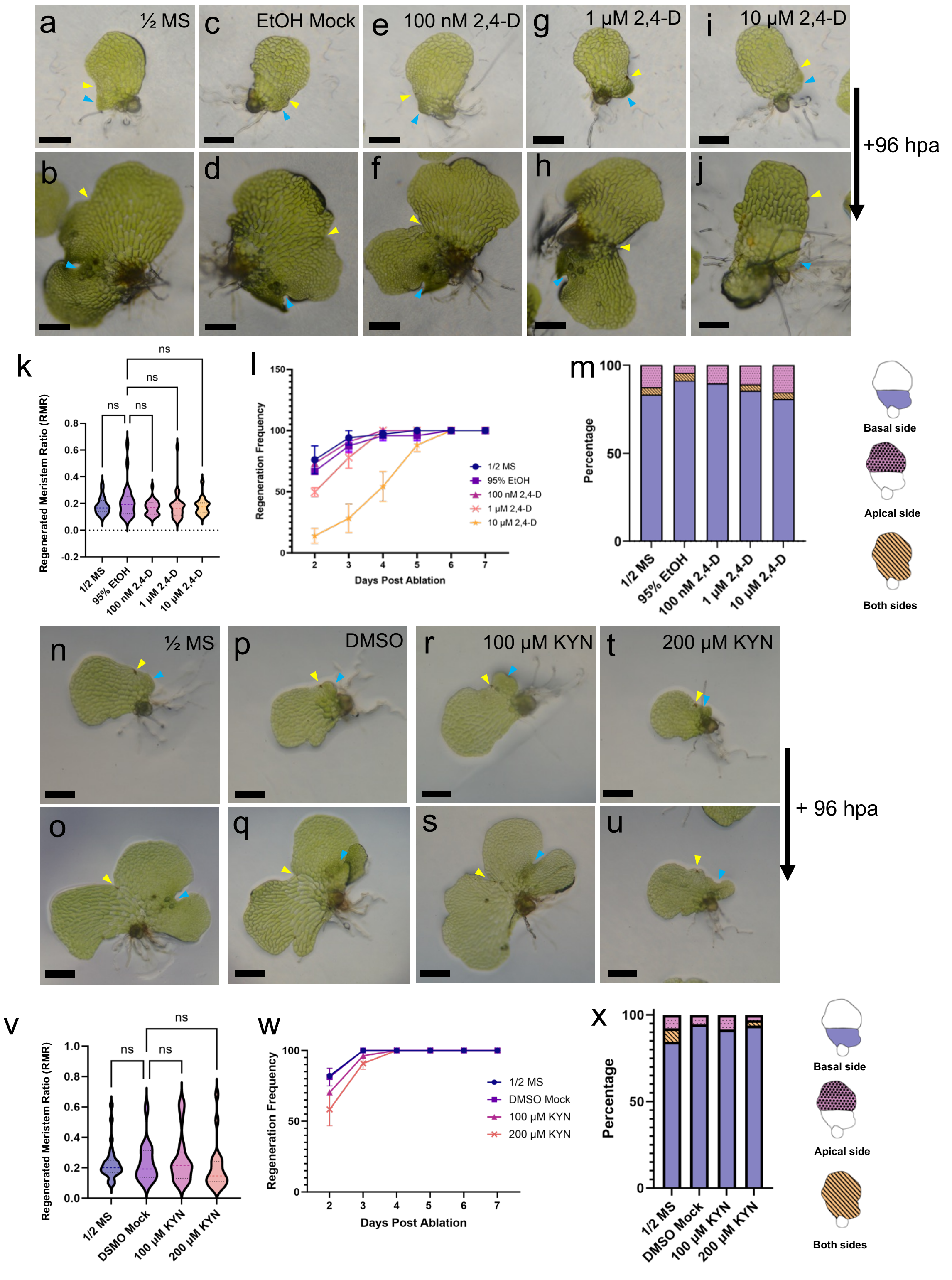

Supplemental Figure S7. Effects of 2,4-D and KYN dosage on the regeneration of ablated gametophytes (a–j,n–u) Live gametophytes on treatment media 2 or 3 dpa and the same individuals 96 h later. (a–m) Effects of 2,4-D dosage during meristem regeneration. (n–x) Effects of KYN dosage during meristem regeneration. (k,v) Violin plot of RMR values for (k) 2,4-D dosage and (v) KYN dosage (n ≥ 21, dashed line = median, dotted lines = upper and lower quartiles, Kruskal–Wallis test with Dunn's multiple comparisons test; ns, not significant). (l,w) Percentage of regenerated individuals from 2- to 7-days post-ablation during treatment with (l) 2,4-D or (w) KYN. (m,x) Percentage of hermaphrodites with a regenerated meristem in the basal (solid), apical (dotted), or both (striped) portions of the prothallus ($n \geq 21$, Fisher's exact test, no symbol, $p > 0.05$). Yellow and blue arrowheads pointing at ablated and regenerated meristems, respectively.

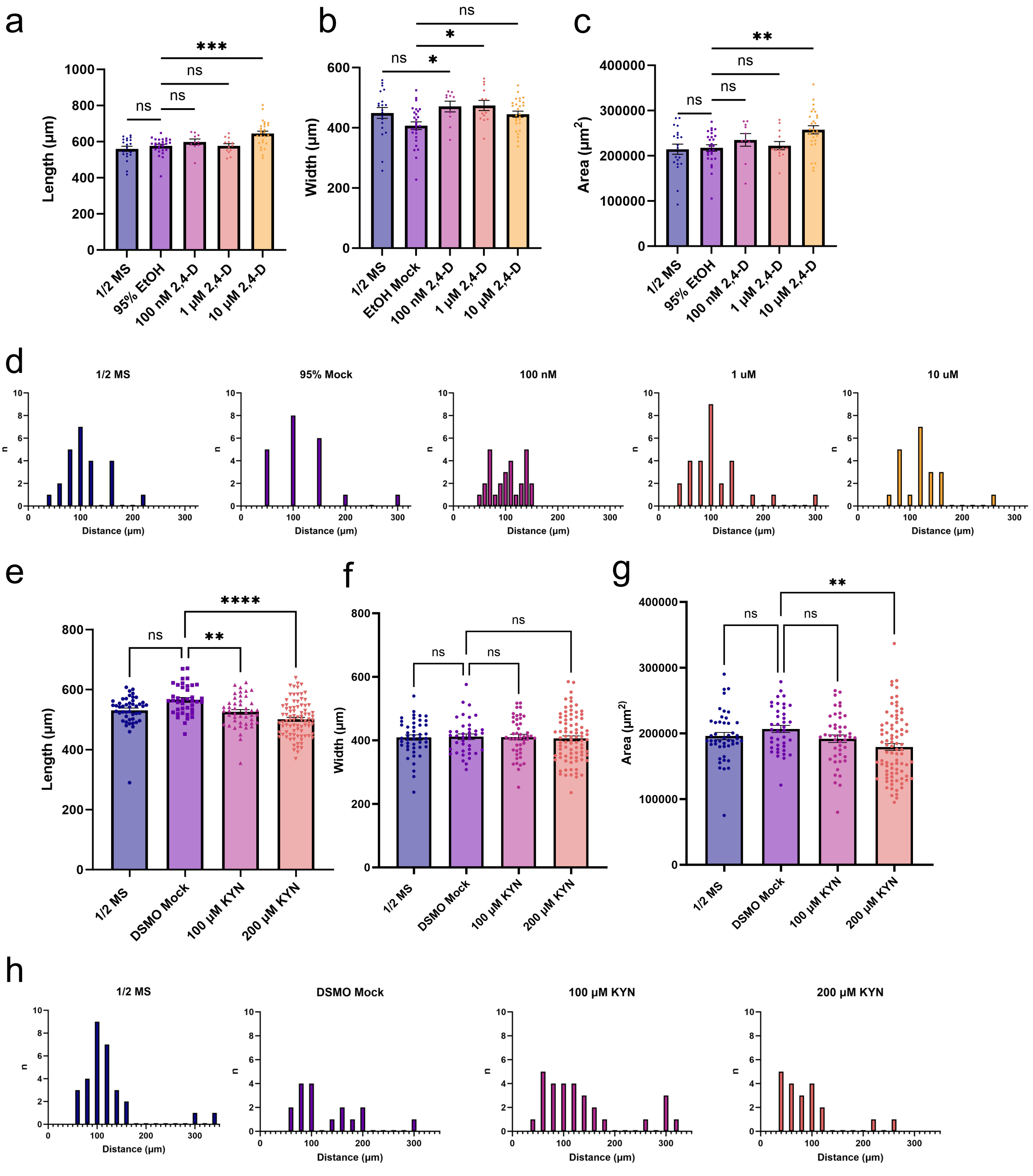

Supplemental Figure S8. Gametophyte shape and raw distance values for 2,4-D and KYN ablated dosage samples (a–c,e–g) Gametophyte shape and size measurements for (a–c) 2,4-D and (e–g) KYN treatments post-ablation. Length, width, and area in µm^2 ($n \geq 40$, mean ± SEM); Kruskal–Wallis with Dunn's comparisons test (KYN—length and area), a one-way ANOVA with Šídák's multiple comparisons test (2,4-D—length, width, and area; KYN—width); *, $p < 0.05$; **, $p < 0.01$; ***, $p < 0.001$; ****, $p < 0.0001$). (d,h) Frequency histograms of raw RMD values for (d) 2,4-D and (h) KYN treatment.
