# Supplementary material for: Auxin Involvement in Ceratopteris Gametophyte Meristem Regeneration
Source: Int J Mol Sci. 2023 Oct 31;24(21):15832. doi: 10.3390/ijms242115832 (PMC10647518; doi:10.3390/ijms242115832)
Supplement: Supplementary file 1 [file ijms-24-15832-s001.zip › Supplemental Table S1 - Primer List.pdf]

**Table S1.** Primers used for cloning, in situ probe synthesis, and RT-qPCR analysis

| **Gene** | **Sequence** | **Use (Product Length in bp)** |
|---|---|---|
| *CrTAA2* | F- CACC*ATGACAACTGCAAGCCCAG<br>R- CTCAGCCCTTTGCTGGCATAATA | Primer Cloning (1211 bp) |
| | F- TGACAACTGCAAGCCCAGGATC<br>R- CCCTCCGTATCCAGCACGTC | RT-qPCR expression analysis (123 bp) |
| | F- CACC*ATGACAACTGCAAGCCCAG<br>R- TAATACGACTCACTATAGGG*CATGTCTCCGTTACCACTACAG | TAA2 antisense probe (297 bp) |
| | F- TAATACGACTCACTATAGGG*ATGACAACTGCAAGCCCAG<br>R- CATGTCTCCGTTACCACTACAG | TAA2 sense probe (297 bp) |
| *CrUBQ* | F-GATGGCCGTACTCTTGCAGAC<br>R-GGAGACGAAGCACGAGATGA | RT-qPCR expression analysis (348 bp) |
| *CrWOXB* | F- CACC*CAACAAGGTGTAGCAAATGG<br>R- TAATACGACTCACTATAGGG*TCAATTTGATGCACGTGGAATG | WOXB antisense probe (272 bp) |
| | F- TAATACGACTCACTATAGGG*CACC*CAACAAGGTGTAGCAAATGG<br>R- TCAATTTGATGCACGTGGAATG | WOXB sense probe (272 bp) |
| *CrTAA1* | F- ATTGCCTCACCCTAGAAAG<br>R- CATGAGCATCGACAAACAG | RT-qPCR expression analysis (196 bp) |
| *CrTAA3* | F- GCAATGCTGTCACTGATGGGTGT<br>R- CAGTACCTGGCTTGATCGTCTTGAC | RT-qPCR expression analysis (177 bp) |
| *CrYUC2-3* | F- GTTAGCATGGCAATATCGTC<br>R- GACCTTCCGCTCTGTATG | RT-qPCR expression analysis (146 bp) |
